# Supplementary material for: Exploring the Interplay between the Hologenome and Complex Traits in Bovine and Porcine Animals Using Genome-Wide Association Analysis
Source: Int J Mol Sci. 2024 Jun 5;25(11):6234. doi: 10.3390/ijms25116234 (PMC11172659; doi:10.3390/ijms25116234)
Supplement: Supplementary file 1 [file ijms-25-06234-s001.zip › Supplementary File S1.pdf]

Supplementary File S1: Figures.

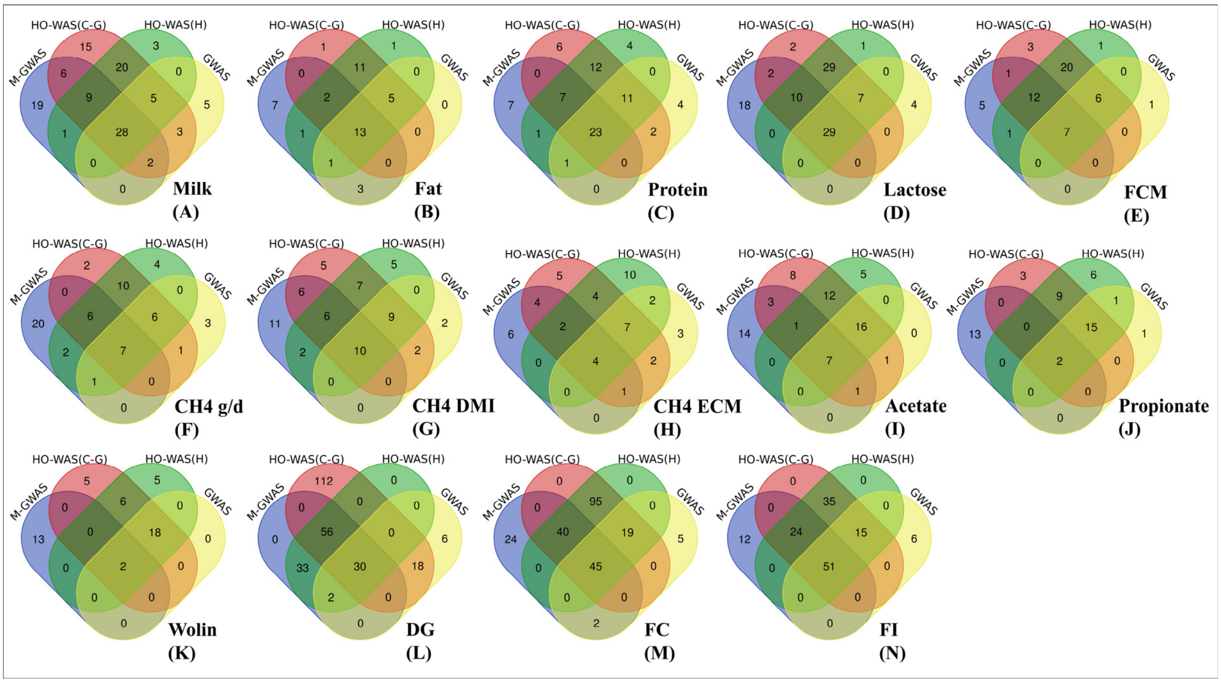

Figure S1. The unique set of genes identified by GWAS, M-GWAS, HWAS-CG, and HWAS-H via TWAS (p_{twas}<0.05) for complex traits. Venn diagram of (A) milk, (B) fat, (C) protein, (D) lactose, (E) FCM, (F) CH4 g/d, (G) CH4 DMI, (H) CH4 ECM, (I) acetate, (J) propionate, (K) wolin, (L) DG, (M) FC, and (N) FI.

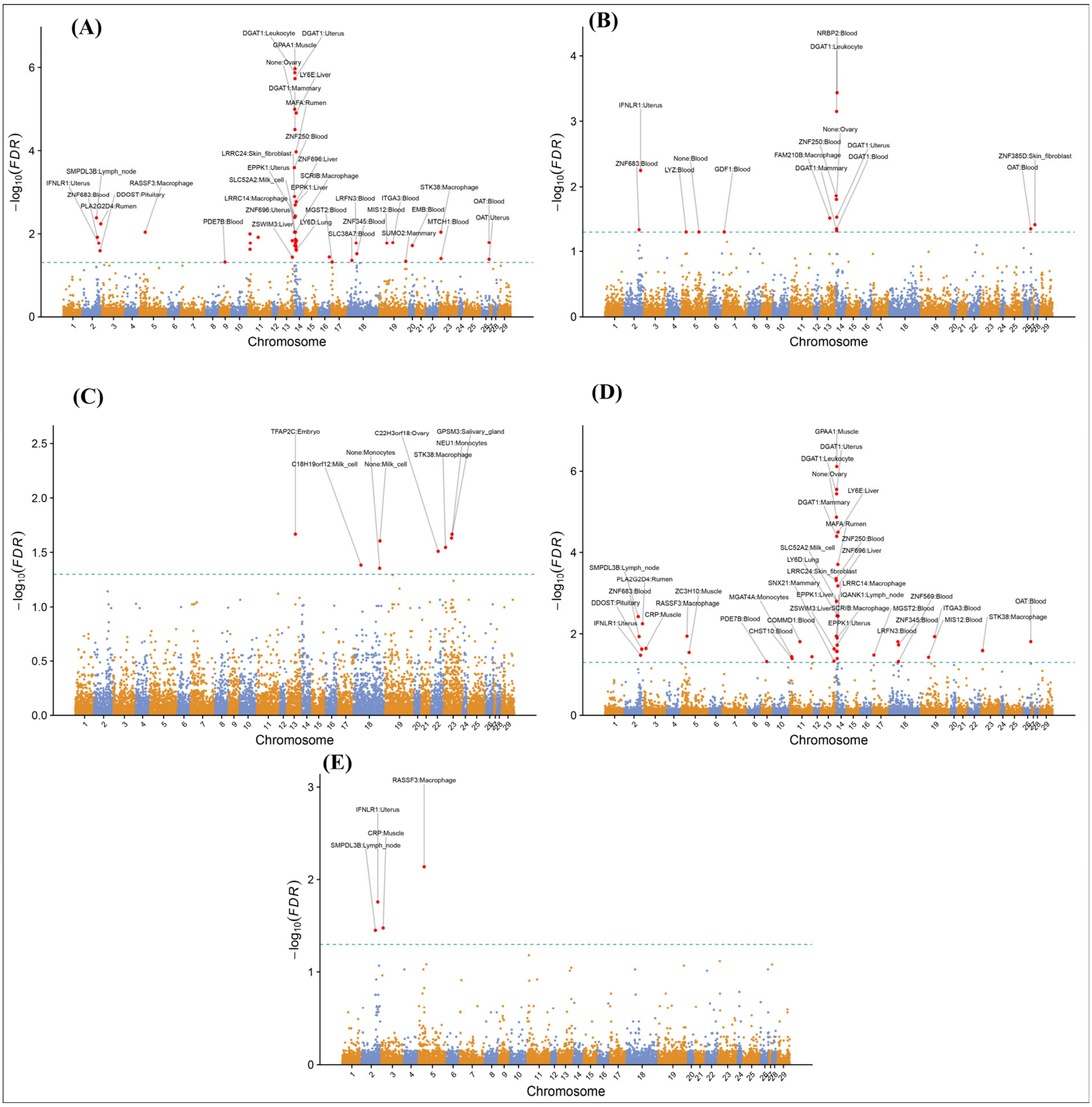

Figure S2. Manhattan plot of TWAS analysis of milk yield traits (FDR < 0.05). (A) Milk, (B) fat, (C) protein, (D) lactose, and (E) FCM.

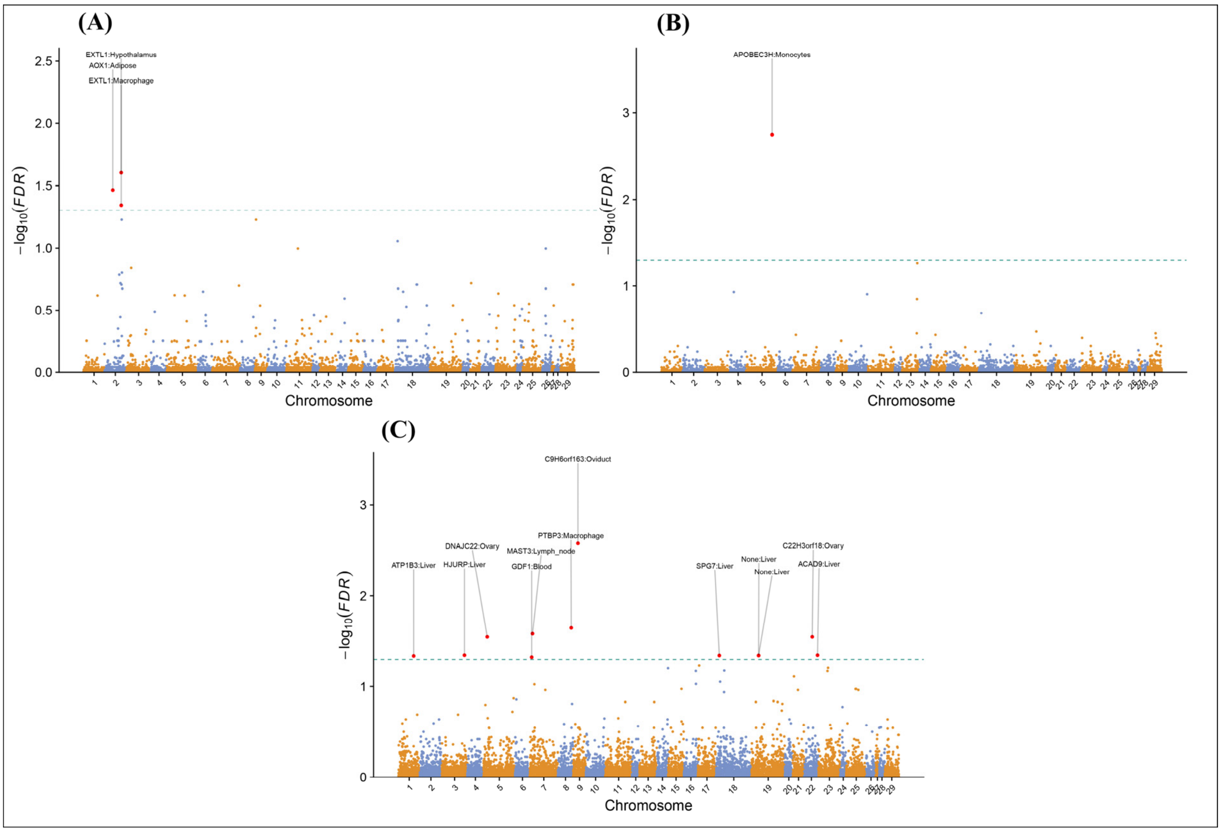

Figure S3. Manhattan plot of TWAS analysis of methane emissions traits (FDR < 0.05). (A) CH4 g/d, (B) CH4 DMI, and (C) CH4 ECM.

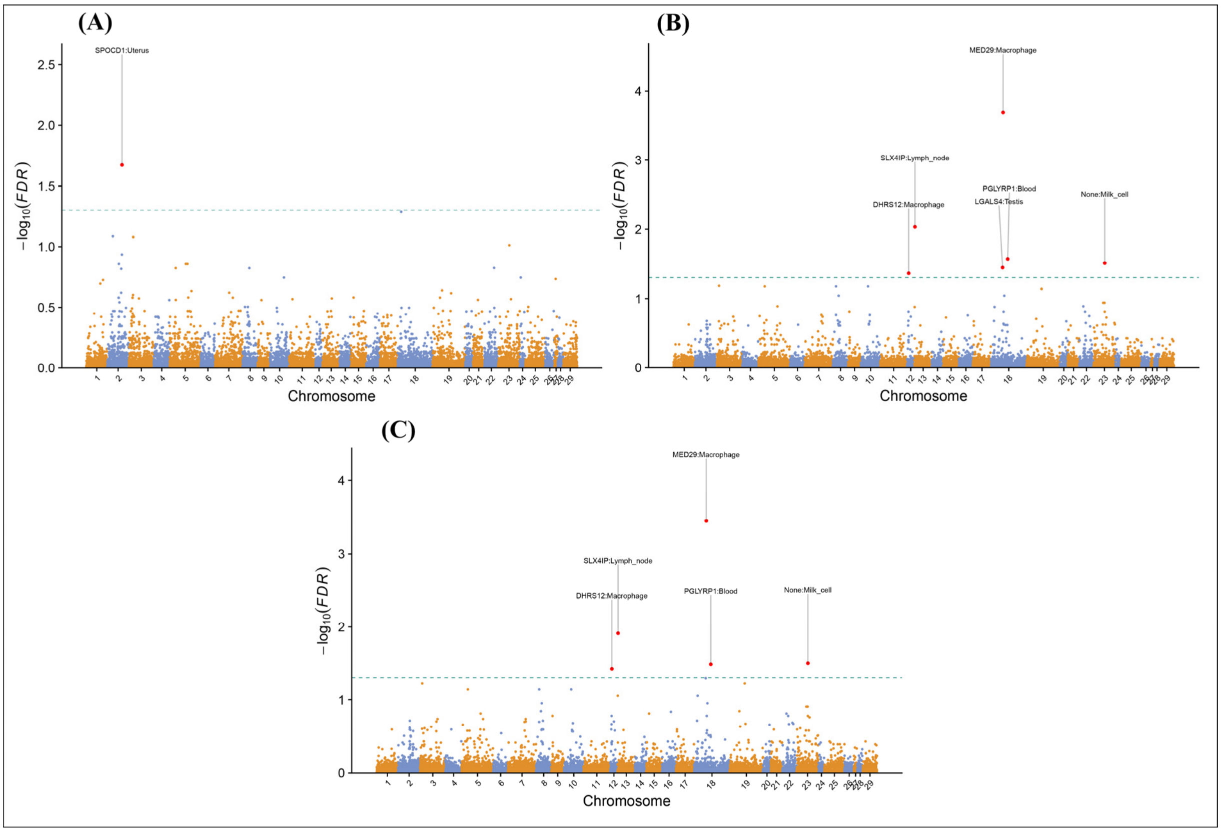

Figure S4. Manhattan plot of TWAS analysis of rumen physiology traits (FDR < 0.05). (A) acetate, (B) propionate, and (C) wolin.

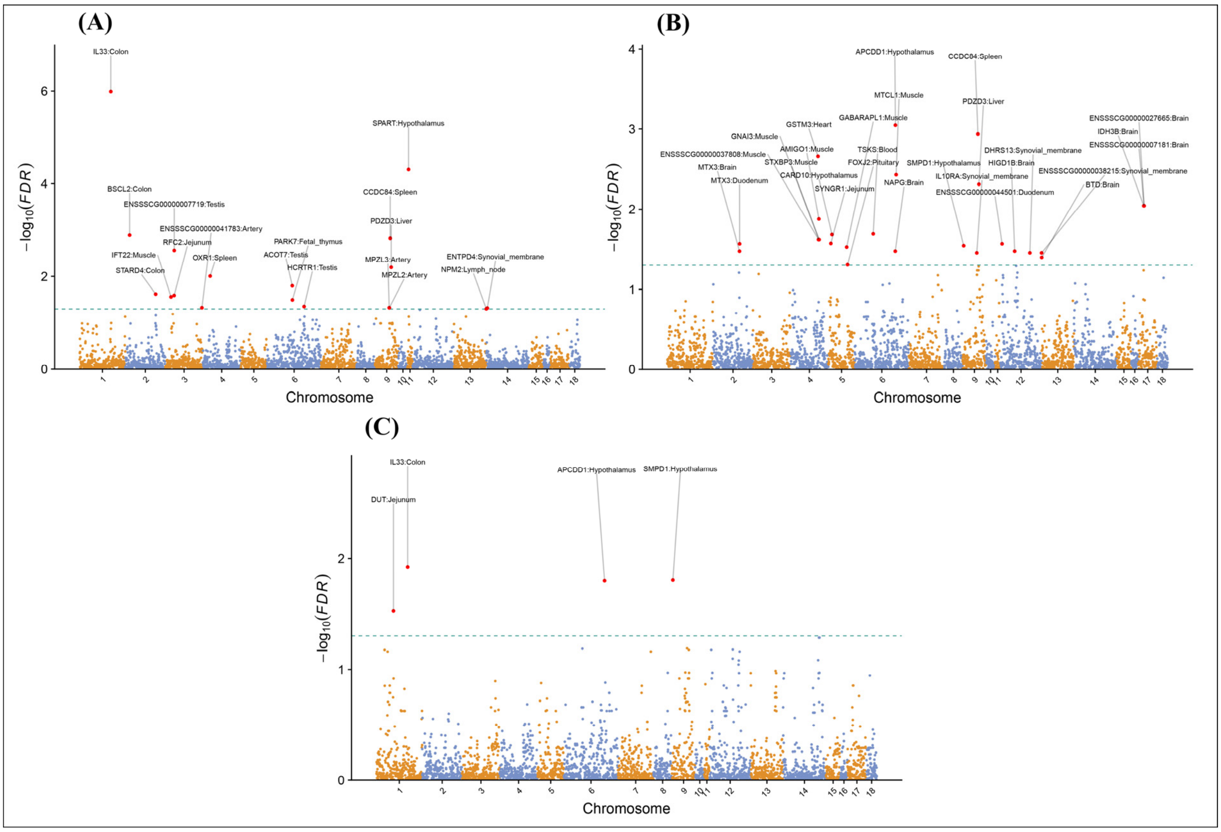

Figure S5. Manhattan plot of TWAS analysis of gut microbial composition traits traits (FDR < 0.05). (A) Daily gain, (B) feed conversion, and (C) feed intake.

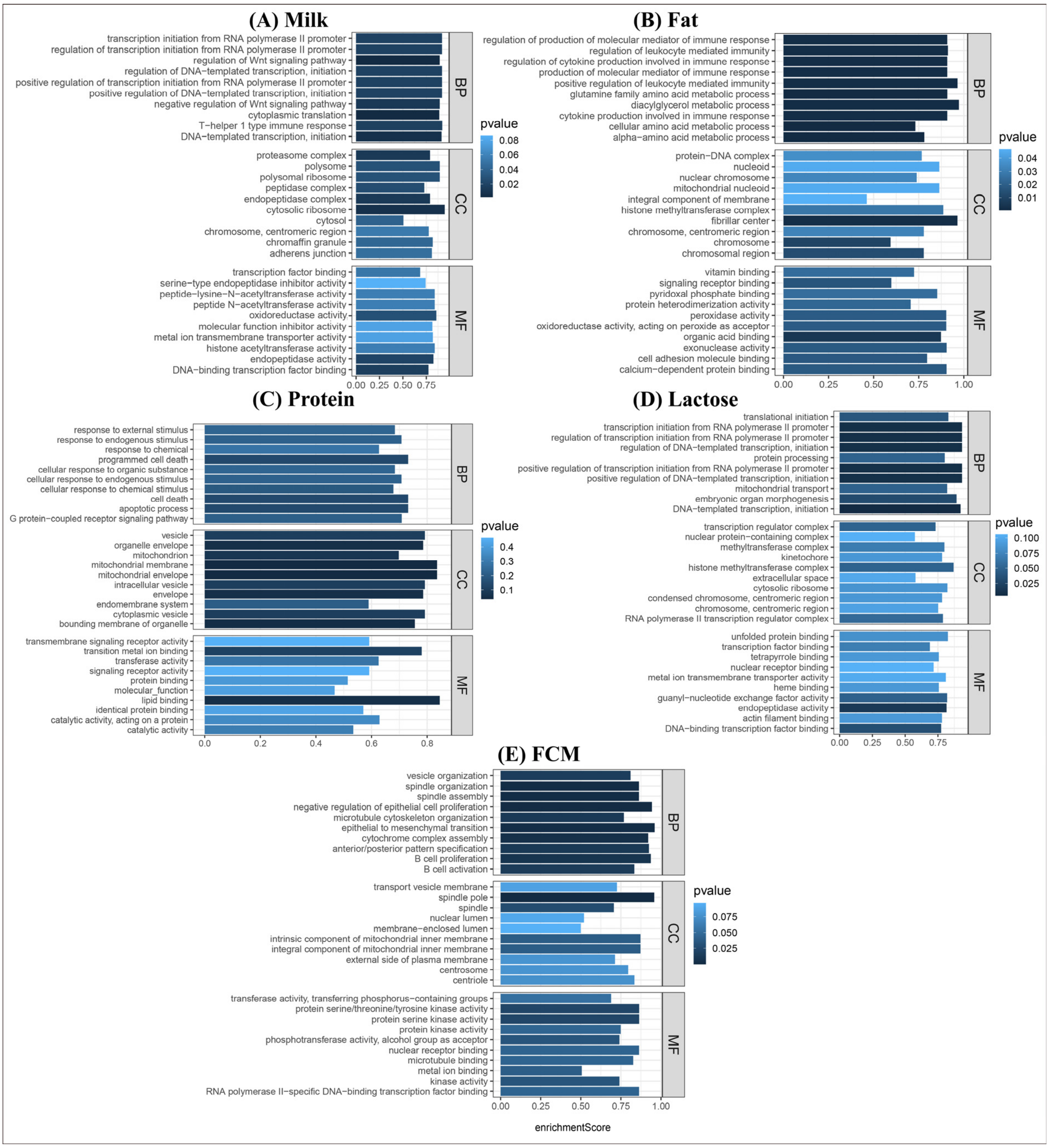

Figure S6. TWAS associated GO enrichment of tissue-specific genes in milk yield. Bar plot of GO terms in (A) milk, (B) fat, (C) protein, (D) lactose, and (E) FCM.

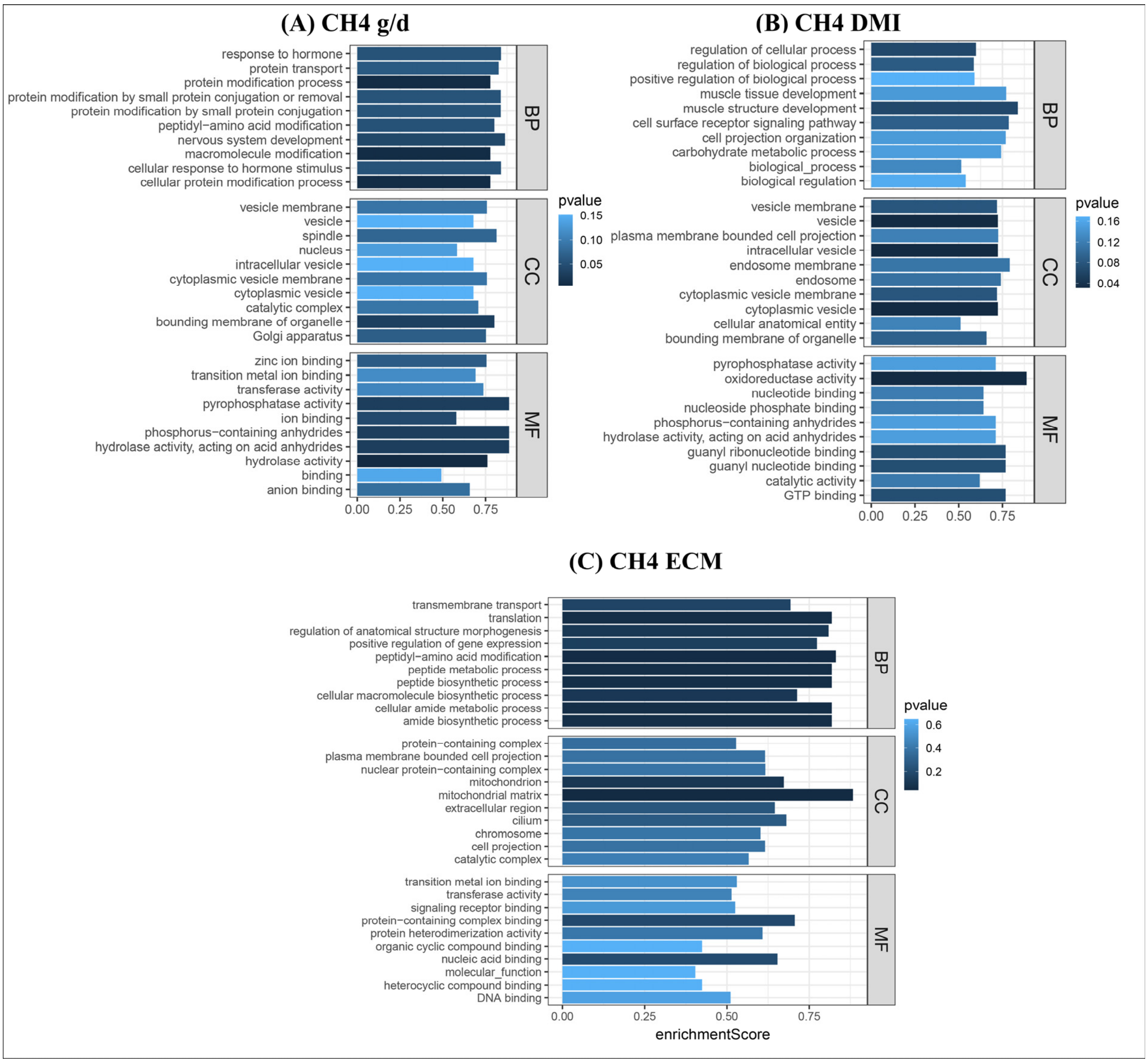

Figure S7. TWAS associated GO enrichment of tissue-specific genes in methane emissions traits.Bar plot of GO terms in (A) CH4 g/d, (B) CH4 DMI, and (C) CH4 ECM.

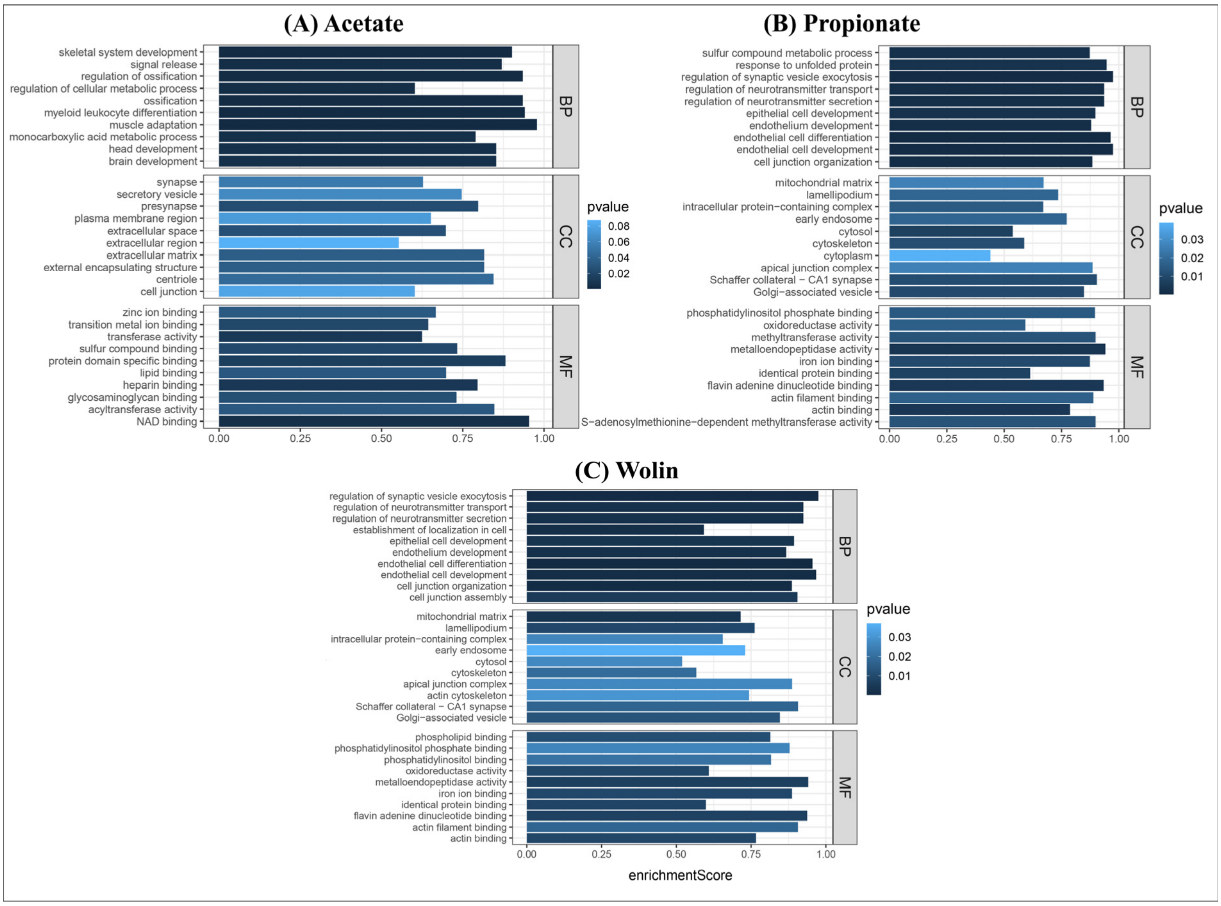

Figure S8. TWAS associated GO enrichment of tissue-specific genes in rumen physiology traits. Bar plot of GO terms in (A) acetate, (B) propionate, and (C) wolin.

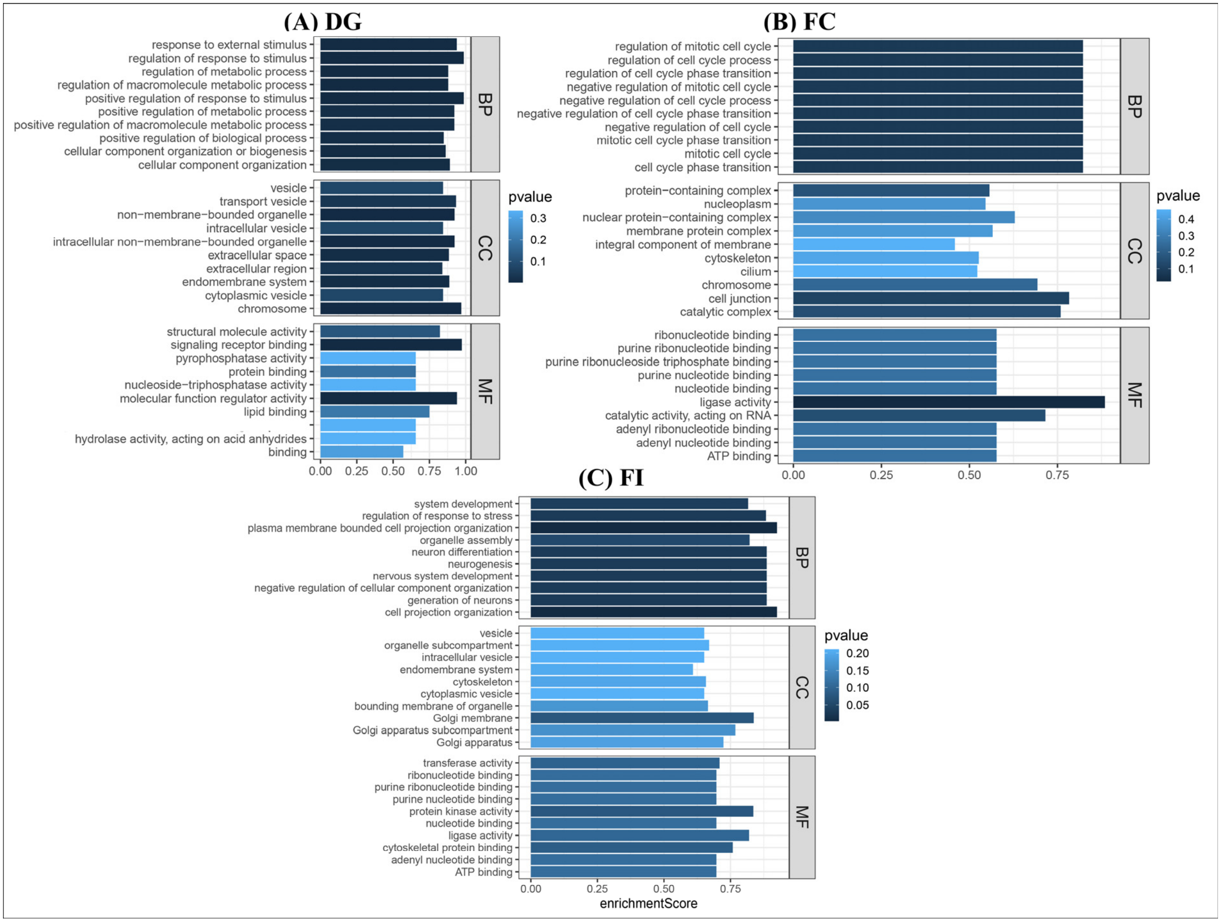

Figure S9. TWAS associated GO enrichment of tissue-specific genes in gut microbial composition. Bar plot of GO terms in (A) DG, (B) FC, and (C) FI.
